# Seed-Specific Expression of a Lysine-Rich Protein Gene, *GhLRP*, from Cotton Significantly Increases the Lysine Content in Maize Seeds

**DOI:** 10.3390/ijms15045350

**Published:** 2014-03-27

**Authors:** Jing Yue, Cong Li, Qian Zhao, Dengyun Zhu, Jingjuan Yu

**Affiliations:** State Key Laboratory of Agrobiotechnology, College of Biological Sciences, China Agricultural University, Beijing 100193, China; E-Mails: yuejinglove@126.com (J.Y.); lnlicong_123@163.com (C.L.); zhaoqian@cau.edu.cn (Q.Z.); zhudy@cau.edu.cn (D.Z.)

**Keywords:** *GhLRP*, lysine-rich protein, lysine, transgenic maize

## Abstract

Maize seed storage proteins are a major source of human and livestock consumption. However, these proteins have poor nutritional value, because they are deficient in lysine and tryptophan. Much research has been done to elevate the lysine content by reducing zein content or regulating the activities of key enzymes in lysine metabolism. Using the naturally lysine-rich protein genes, *sb401* and *SBgLR*, from potato, we previously increased the lysine and protein contents of maize seeds. Here, we examined another natural lysine-rich protein gene, *GhLRP*, from cotton*,* which increased the lysine content of transgenic maize seeds at levels varying from 16.2% to 65.0% relative to the wild-type. The total protein content was not distinctly different, except in the six transgenic lines. The lipid and starch levels did not differ substantially in *Gossypium hirsutum* L. lysine-rich protein (GhLRP) transgenic kernels when compared to wild-type. The agronomic characteristics of all the transgenic maize were also normal. *GhLRP* is a high-lysine protein candidate gene for increasing the lysine content of maize. This study provided a valuable model system for improving maize lysine content.

## 1. Introduction

Maize (*Zea mays* L.) is a major source of food and animal feed worldwide, especially in developing and underdeveloped countries. However, maize seeds are deficient in two essential amino acids: lysine and tryptophan [1]. Essential amino acids are necessary for good health, but must be obtained from the diet, because humans and monogastric animals cannot synthesize them. Consuming a protein-deficient diet over the long term may lead to poor growth, disease and, in severe cases, death [2,3]. Therefore, improving the composition of maize proteins is highly desirable.

A number of maize opaque mutants with increased lysine content have been characterized [4]. The *opaque-2* (*o2*) mutant contains over 70% more lysine than normal seeds [5]. The *o2* gene encodes an endosperm-specific basic leucine zipper (b-ZIP) transcription factor that regulates the transcription of genes encoding 22-kDa α-zeins and other proteins, such as the cytoplasmic pyruvate orthophosphate dikinase (CyPPDK1), the lysine-ketoglutarate reductase/saccharopine dehydrogenase (LKR/SDH), involved in lysine catabolism, the b-32 ribosome-inactivating protein and the protein synthesis elongation factor (eEF1A). A significant decrease in 22-kDa α-zeins results in increased lysine content. The loss of LKR activity leads to increased levels of free lysine. Moreover, the increased eEF1A, which has a high (10% *w*/*w*) lysine content, contributes most to the increased levels of lysine in the *o2* mutant [6].

Unfortunately, the original *o2* mutant is associated with inferior agronomic traits, including lower yields and a soft, floury kernel that makes it more susceptible to insects, pathogens and mechanical damage [7,8]. To overcome these defects while maintaining superior nutritional quality, *o2* endosperm modifier genes were introduced into *o2* mutants, and quality protein maize (QPM) varieties were developed [9–13]. Some released QPM varieties, such as T × 802, possess desirable agronomic characteristics and have 50% more lysine content than standard non-QPM lines [14]. However, the lengthy process (~30 years) of QPM breeding indicates the limited feasibility of conventional approaches to increase the lysine content of maize [15].

Recent advances in plant genetic engineering have provided new opportunities to improve the lysine content of maize. Attempts have been made to reduce the levels of some lysine-poor seed storage proteins, such as α- and β-zein, by RNAi [1,16–18]. Unfortunately, some of these transgenic plants exhibit opaque phenotypes [16,17]. As research on lysine metabolism continues, some key auxiliary enzymes, such as aspartate kinase (AK), dihydrodipicolinate synthase (DHDPS) and lysine LKR/SDH, have also been studied to increase the lysine levels in maize seeds [1,19–22]. However, only free lysine was increased in these transgenic plants [22].

Wild-type genes artificially mutated to increase the number of lysine codons or synthetic genes encoding lysine-rich proteins have been transformed into maize [23]. However, most of these attempts were unsuccessful, because these inherently-unstable proteins did not accumulate to sufficient levels in maize endosperm [24,25]. Alternatively, heterologous expression of lysine-rich proteins has significantly increased lysine and total protein levels. For example, Bicar *et al*. introduced a construct containing a milk protein gene under the control of the seed-specific 27-kDa γ-zein promoter into the maize genome, and the transgenic lines had 29%–47% more lysine in the endosperm, although total protein content was not significantly altered [26]. In addition, Yu *et al*. and Lang *et al*. showed that seed-specific expression of the lysine-rich cytoskeleton-associated protein genes, *sb401* and *SBgLR*, significantly increased lysine and total protein contents in maize seeds, respectively [27,28]. Furthermore, Tang *et al*. conducted a nutritional assessment of transgenic *sb401* maize and showed that transgenic maize seeds had significantly higher levels of total protein, lysine, other amino acids, several minerals and vitamin B_2_ than did conventional QPM [29].

In this study, a lysine-rich protein gene, *GhLRP*, was cloned from the cotton genome (*Gossypium hirsutum* L.). The GhLRP protein is a water-soluble protein with a high lysine content (18.97% *w*/*w*). In addition, the GhLRP protein is rich in glutamic acid and contains repeated sequences. So far, very little is known about its function.

To investigate whether the accumulation of GhLRP in maize kernels could increase lysine content, a construct containing the *GhLRP* gene under the control of the seed-specific promoter, F128, was introduced into the maize genome by *Agrobacterium*-mediated transformation. The F128 promoter is a seed-specific promoter from foxtail millet (*Setaria italica*) (China (CN) 101063139A). Ma *et al*. showed that the activity of the F128 promoter is higher than that of the maize 19-kDa α-zein promoter [30]. We quantified the lysine content of transgenic maize lines expressing GhLRP, and the results showed that transgenic lines contained a higher level of lysine.

## 2. Results

### 2.1. Gene Cloning and Plasmid Construction

The *GhLRP* gene was cloned from the cotton genome. It consists of only one exon encoding a protein with 195 amino acids (Figure S1A,B). Using DNAMAN software 6.0.3.48 (Lynnon Corporation, Vaudreuil-Dorion, QC, Canada), we analyzed the GhLRP, and the result showed that GhLRP was a water-soluble protein with a deduced molecular weight of 24-kDa and a pI of 6.2. It was rich in lysine and glutamic acid (Figure S1C).

**Figure 1.**
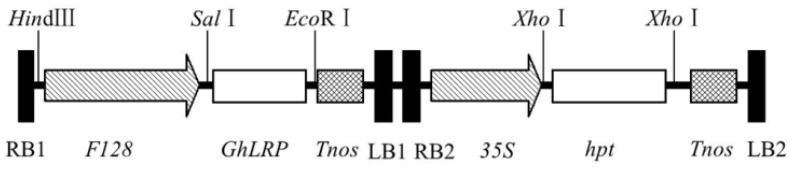
Schematic diagram of binary vector pSB130-GhLRP developed for maize transformation. The plasmid consisted of two T-DNAs. The right and left borders of the two T-DNAs are designated as RB1, LB1 and RB2, LB2, respectively. T-DNA1 contains the *GhLRP* expression cassette, in which the *GhLRP* gene is regulated by the promoter, F128, and the Nos terminator, and T-DNA2 includes the selectable marker gene, *hpt*, expression cassette controlled by the CaMV 35S promoter and the Nos terminator.

To construct the expression vector, *GhLRP* was sub-cloned into the binary vector, pSB130-SBgLR, with-twin T-DNA. The finished plasmid was named pSB130-GhLRP and carried two independent T-DNAs. One contained the *GhLRP* expression cassette, in which the *GhLRP* gene was driven by the promoter, F128, to achieve seed-specific expression, and the other included the selectable marker gene, *hpt*, expression cassette controlled by the CaMV 35S promoter (Figure 1).

### 2.2. Plant Transformation and Molecular Analyses

**Figure 2.**
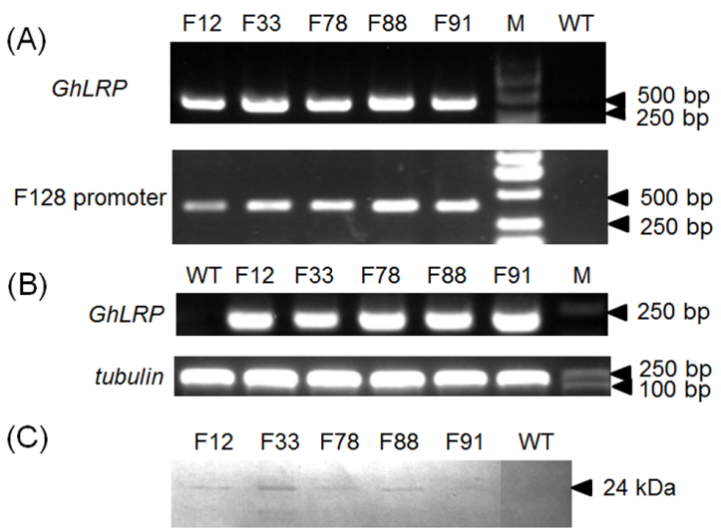
Molecular analyses of several T_1_ transgenic events. (**A**) PCR analysis of five transgenic lines. PCR was performed using primers corresponding to the *GhLRP* sequence (top) and the promoter F128 sequence (bottom). M, DNA marker; WT (wild-type): the segregating populations of hybrid 08 × 178 in T_1_ were used as a negative control; (**B**) Reverse transcription (RT)-PCR analysis. The maize *tubulin* gene (accession number AY103544) was used as the internal control; (**C**) Western blot analysis.

The construct pSB130-GhLRP was transformed, mediated by *Agrobacterium tumefaciens* LBA4404, into immature embryos (1.5–2.0 mm) of the hybrid 08 × 178 at 10–12 days after pollination (DAP). The processes of maize transformation and plant regeneration are shown in Figure S2. Using PCR amplification with primers corresponding to the *GhLRP* and promoter F128 sequences, respectively, a total of 88 T_0_ individual transgenic lines were identified (data not shown). The T_0_ transgenic plants were self-pollinated to obtain T_1_ progeny. A sample of the PCR results of the T_1_ transgenic lines is shown in Figure 2A. To test whether *GhLRP* was transcribed in the transgenic lines, reverse transcription (RT) PCR was conducted. An RT-PCR product of the expected size (163 bp) was detected from the cDNA of T_1_ transgenic immature kernels (20 DAP), but not from the wild-type (WT) (Figure 2B). Additionally, western blot analysis was performed using GhLRP antiserum to test for the expression of the GhLRP. As a result, the GhLRP protein band was detected in T_1_ mature kernels, while no bands were present in WT kernels (Figure 2C). The molecular mass of the specific bands on SDS-PAGE was 24-kDa, consistent with the size of the deduced protein. These results demonstrated that not only was *GhLRP* integrated into the maize genome, the GhLRP protein also accumulated in mature seeds.

### 2.3. Lysine and Total Protein Contents in Transgenic Kernels

Using the ninhydrin and Kjeldahl methods, we analyzed the lysine and total protein contents of 88 T_1_ transgenic lines. The lysine contents of 40 transgenic lines were increased relative to WT by levels ranging from 16.2% to 65.0%. In particular, 26 transgenic lines had significantly more lysine than WT (Table 1). The total protein content was not distinctly different, except in the F12, F45, F47, F78, F87 and F88 lines. To meet the demands of breeding and to test the heritability of increased lysine content, the progenies of the F12, F78 and F88 lines were analyzed in the T_2_, T_3_ and T_4_ generations. We found that the lysine and protein contents of all three lines were increased significantly relative to WT in the following generations (Table 2). The lysine contents of F12, F78 and F88 transgenic lines were increased by 50.0%, 46.2% and 57.7%, respectively, in T_2_, by 23.3%, 30.0% and 36.7%, respectively, in T_3_ and by 22.6%, 29.0% and 41.9%, respectively, in T_4_.

**Figure 3.**
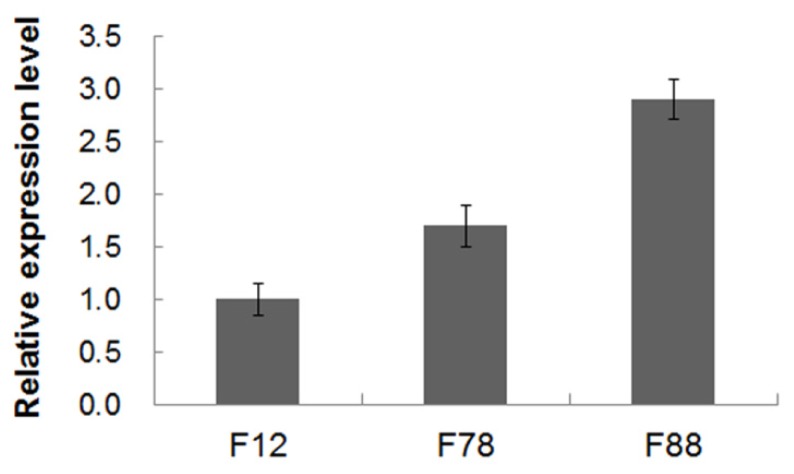
Relative expression levels of *GhLRP* in T_3_ kernels of three transgenic lines. The transcriptional levels of *GhLRP* in F12, F78 and F88 transgenic lines (20 days after pollination) in T_3_ were quantified by real-time PCR. The maize *tubulin* gene was used as a normalization control.

Further, the transcriptional levels of *GhLRP* in these three transgenic lines were quantified by quantitative real-time PCR (qRT-PCR). Variation in the relative expression levels of *GhLRP* was observed among the three transgenic lines in the T_3_ generation. The transcriptional level of *GhLRP* in T_3_ kernels of the F78 line was slightly higher than that in the F12 line, while the level was highest in the F88 line (Figure 3).

### 2.4. Starch and Lipid Contents in Transgenic Kernels

Protein, lipid and starch are the major seed storage components in the endosperms of maize kernels. In conventional maize, lipid and starch contents decrease as protein content increases. When we analyzed the lipid and starch contents of the F12, F78 and F88 transgenic lines, we found no obvious differences from WT kernels (Table 3).

### 2.5. Zein and Non-Zein Proteins Accumulation in Transgenic Kernels

**Figure 4.**
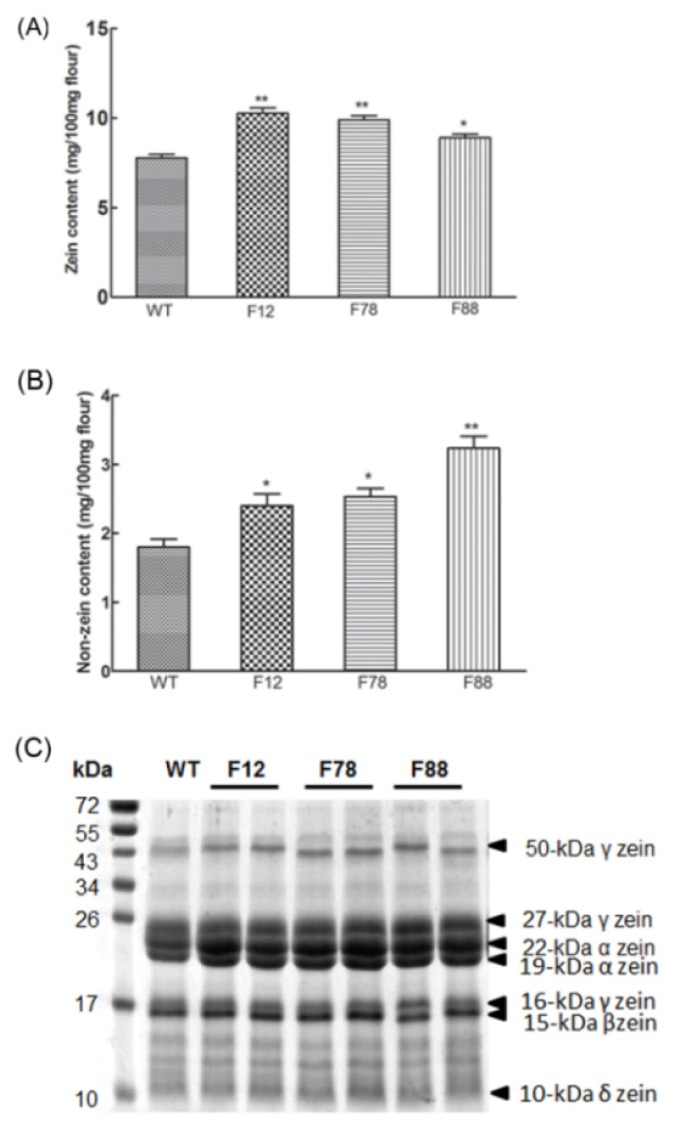
Zein and non-zein accumulation pattern in T_2_ kernels of three transgenic lines. (**A**) Zein content; (**B**) non-zein content. All the calculations were performed with technical triplicates and biological triplicates, and 20 mature kernels were used per assay. The Student’s *t-*test is used to evaluate the difference between the transgenic maize and WT (* *p* < 0.05; ** *p* < 0.01); (**C**) Zein proteins of WT, F12, F78 and F88 lines were analyzed by SDS-PAGE. For SDS-PAGE, 5 μL samples taken from the same amount of mature kernel flour were loaded to 15% polyacrylamide gel. The size for each protein marker and sample band was indicated by the numbers in the “kDa” columns.

The storage proteins in maize endosperm are categorized into albumins, globulins, glutelins and zeins based on their solubilities in different solvents [31]. Zeins are the main storage proteins and usually account for 50%–70% of the endosperm storage proteins. To further identify the content of each protein fraction in the F12, F78 and F88 transgenic lines, zein and non-zein proteins were extracted and quantified. The T_2_ F12, F78 and F88 kernels contained significantly more zeins, with averages of 10.3, 9.9 and 8.9 mg/100 mg of flour, respectively, than did WT (7.8 mg/100 mg of flour) (Figure 4A). The content of non-zein proteins in F12, F78 and F88 was 2.4, 2.5 and 3.2 mg/100 mg of flour, respectively, *versus* 1.8 mg/100 mg of flour in WT (Figure 4B). Furthermore, the protein profiles of zeins in WT, F12, F78 and F88 mature kernels were examined by SDS-PAGE. The levels of 19 and 22-kDa α-zeins and 27 and 50-kDa γ-zeins were higher in the three transgenic lines (Figure 4C). Similarly, the zein and non-zein contents of T_4_ transgenic kernels were much higher than that of WT (Figure S3). However, further experiments are needed to determine which kinds of non-zeins were affected.

### 2.6. Agronomic Quality Analysis

**Figure 5.**
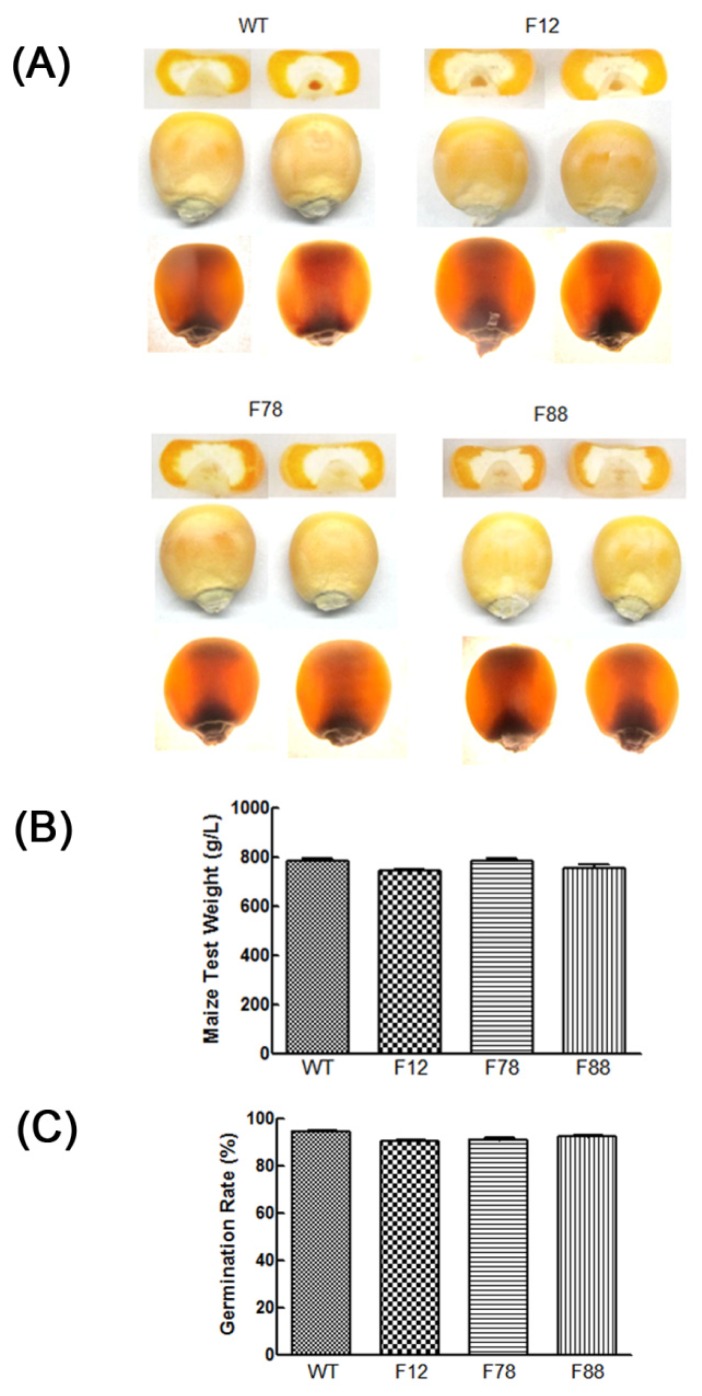
Kernel appearance and maize test weight of T_3_ seeds in three transgenic lines. (**A**) Kernel appearance. Three kernels were selected at random from WT and three transgenic lines, and their appearances were observed with an incandescent (top and middle) and transmitted (bottom) light; (**B**) Maize test weight; (**C**) Germination rate. A Student’s *t-*test was used to evaluate the differences between each transgenic line and WT.

To investigate whether the *GhLRP* transgene affected the agronomic traits of transgenic maize, some morphological features of WT and F12, F78 and F88 transgenic maize in the T_2_ and T_3_ generations were analyzed (Table 4 and Table S1). Differences in plant height, ear height, ear length, bald tip length, ear diameter, numbers of ear rows and kernel weight were not significant between the transgenic and WT maize. Kernel hardness is a critical agronomic trait in maize. We estimated the kernel appearance and test weight of mature kernels from the transgenic lines and WT, and no obvious differences were found; all the seeds were vitreous kernels (Figure 5A,B). Furthermore, data from our field tests showed that the germination rates of T_3_ seeds of all three lines were also similar to those of WT (Figure 5C). Our results indicated that the *GhLRP* transgene did not sufficiently disturb the expressions of native genes to damage agronomic quality.

## 3. Discussion

The objective of our study was to increase the lysine content of maize seeds by expressing the high-lysine protein GhLRP in transgenic maize kernels. The average lysine content of WT kernels in T_1_ generation was 0.28 g/100 g seed, while the average lysine contents of 26 transgenic lines were from 0.35 to 0.46 g/100 g seed (Table 1), which indicated that expression of GhLRP might cause significant increases in lysine content in transgenic maize. Moreover, the high lysine contents of F12, F78 and F88 transgenic maize were heritable in the T_2_, T_3_ and T_4_ generations. The lysine content was increased by 50.0%, 23.3% and 22.6% in T_2_, T_3_ and T_4_ kernels of F12, respectively, by 46.2%, 30.0% and 29.0% in T_2_, T_3_ and T_4_ kernels of F78, respectively, and by 57.7%, 36.7% and 41.9% in T_2_, T_3_ and T_4_ kernels of F88, respectively. Further, in these lines, lysine content was positively correlated with the transcriptional levels of *GhLRP*; the transgenic line with the highest *GhLRP* transcript level also contained the most lysine (Figure 3 and Table 2). These results demonstrated that *GhLRP* could increase the lysine content of transgenic maize.

Several studies have reported the use of lysine-rich proteins to increase lysine content in maize. The expression of a milk protein resulted in significant increases in lysine content in transgenic maize kernels; ELISA analysis suggested that the accumulation of this protein explained the amino acids changes [26]. Moreover, the milk protein might affect gene expression and/or metabolism, resulting in increased expression of high-lysine proteins or the production of elevated levels of free lysine [26]. In our previous work, overexpression of *sb401* or SBgLR significantly increased lysine content in transgenic maize seeds [27,28]. The *sb401* gene encodes a lysine-rich protein named LRP. The accumulation of LRP or SBgLR, together with increases in some lysine-rich non-zeins, might be the major reasons for the lysine increase [27,32]. The GhLRP protein is similar to the SBgLR protein, which is also rich in lysine and glutamic acid, and contains repeated sequences. The expression of GhLRP resulted in significant increases in lysine content in transgenic maize. The accumulation of GhLRP may contribute directly to the high lysine content. However, it is not sufficient to explain the observed changes in lysine content. A second possibility is that GhLRP caused the upregulation of high-lysine proteins that resulted in the high lysine content of transgenic maize. We are currently examining the function of GhLRP to determine how the *GhLRP* gene increases lysine content.

To obtain high-lysine corn with good characteristics, we estimated the agronomic and quality traits of transgenic maize. Significant differences in kernel appearance, kernel weight, test weight and germination rate were not found between transgenic maize and WT (Tables 4 and S1 and Figure 5). Moreover, there were no significant drawbacks in terms of total protein, lipid or starch contents in most of the transgenic lines (Tables 1 and 3). While the *opaque-2* mutant contains elevated levels of lysine and tryptophan, its soft, floury endosperm and reduced yield and protein content prevented its acceptance by farmers in both the developed and developing world [33]. Our results suggested that the *GhLRP* transgene did not damage the agronomic and quality traits of maize.

The transcriptional levels of *GhLRP* detected in the endosperm varied among transgenic lines (Figure 3). These differences may be caused by differences in transgene insertion location and/or copy number [26]. In our study, immature embryos of the 08 × 178 hybrid were used for transformation, resulting in a mixture of homozygous and heterozygous kernels in early generations. The gene dosage effect may result in homozygous kernels having more GhLRP than heterozygous kernels. If this is true, the lysine content may be higher than reported here. We are trying to obtain homozygous maize to improve lysine levels even further.

## 4. Experimental Section

### 4.1. Plant Materials and Growth Conditions

Maize inbred lines 08 and 178 were selected for maize transformation. These two lines were cultivated by Chinese breeders. The origin of inbred line 08 is Huang C. In China, The inbred line Huang C is one of the four major inbred maize lines. The origin of inbred line 178 is American hybrid line 78,599. The inbred line 178 is a member of a popular heterotic group used in China. Moreover, the inbred lines Huang C and 178 are the parents of another hybrid ND108 that is also widely grown in China.

Maize inbred lines 08 and 178 were grown in a test field at China Agricultural University (Beijing, China). The inbred maize lines 08 and 178 were cross-pollinated to produce the hybrid line 08 × 178. Immature embryos (1.5–2.0 mm) of this hybrid line at 10–12 DAP were used for *Agrobacterium*-mediated transformation.

### 4.2. Plasmid Construction

The *GhLRP* gene was cloned from the cotton genome (*Gossypium hirsutum* L.), then the cloned *GhLRP* gene was sub-cloned into the *Sal*I-*Eco*RI site of pSB130-SBgLR to replace the *SBgLR* fragment. The binary vector, pSB130-SBgLR, was derived from the double T-DNA binary vector, pSB130 (kindly provided by Shiwen Xin, The Chinese University of Hong Kong, (Hong Kong, China). In this construct, the GhLRP coding sequence was transcriptionally fused to the F128 promoter and the *nos* region of T-DNA1, and the selectable marker gene, *hpt*, driven by the CaMV 35S promoter, was contained in T-DNA2 of this standard binary vector. The expression vector was named pSB130-GhLRP (Figure 2). For plant transformation, this plasmid was transferred into *A. tumefaciens* strain LBA4404.

### 4.3. Maize Transformation

*Agrobacterium*-mediated transformation was performed according to the method of Frame, with some modifications [34]. For infection, 1 mL of *Agrobacterium* suspension (OD_550_ = 0.3–0.4) was added to the embryos. After infection, embryos were transferred to the surface of co-cultivation medium and incubated in the dark at 20 °C for 3 days. They were then transferred to resting medium at 28 °C in the dark for 7 days and subsequently to selection medium containing 5 mg/L hygromycin (Amresco, Solon, OH, USA) for 2 weeks. They were then sub-cultured at 2-week intervals on 10, 15 and then 20 mg/L hygromycin. Finally, the hygromycin-resistant calli were recovered and cultivated in the light, resulting in the generation of T_0_ plants.

### 4.4. PCR Analysis

From each generation, genomic DNA was extracted from fresh leaf tissue using SDS extraction buffer. PCR was carried out using 50 ng DNA, 10 μL of 2× PCR mix buffer (GenStar, Beijing, China) and 0.5 μL of each primer (10 μmol/L) in a 20 μL volume. The forward (5′-GGTAGCCACACCGTCTCGTC-3′) and reverse (5′-TTCTTTCTCATACTCTTTGTGCTCC-3′) primers corresponding to the *GhLRP* sequence amplified a fragment of 420 bp. The PCR conditions were a hot-start for 5 min at 94 °C; 35 cycles of 30 s at 94 °C, 30 s at 60 °C and 30 s at 72 °C; and 10 min at 72 °C. The forward (5′-ATCCGTTGTCATAAATGTGTAG-3′) and reverse (5′-GAGTTTTCTGAGAATGGTACCC-3′) primers corresponding to the F128 promoter sequence amplified a fragment of 378 bp. The PCR conditions were a hot-start for 5 min at 94 °C; 35 cycles of 30 s at 94 °C, 30 s at 56 °C and 30 s at 72 °C; and 10 min at 72 °C. PCR products were separated on 1% (*w*/*v*) agarose gels, stained with ethidium bromide and photographed.

### 4.5. RNA Extraction and RT-PCR and qRT-PCR Analyses

Total RNA of immature endosperm at 20 DAP was isolated by TRIzon (CWBIO, Beijing, China). Residual DNA contaminants were removed with an RNase-free DNase I (TaKaRa, Otsu, Japan). About 2 μg of total RNA was reverse-transcribed to cDNA using the Reverse Transcription System (Promega A3500, Fitchburg, WI, USA), according to the manufacturer’s protocol. RT-PCR was performed using 1 μL cDNA, 10 μL of 2× PCR mix buffer (GenStar, Beijng, China) and 0.5 μL of each primer (10 μmol/L) in a 20 μL volume. Specific forward (5′-CCAAGCTTCTCGTCAACGACTCGACATTTA-3′) and reverse (5′-CGGAATTCCTTTCCTCCTTGTGCATTTCAG-3′) primers were used to amplify a 163-bp internal fragment of *GhLRP*. Additionally, forward (5′-GAGCATGGCATTCAGGCTGACG-3′) and reverse (5′-TCAACAAAAACAGCACGGGGCA-3′) primers were designed to amplify a region of the tubulin transcript as an internal control to ensure the quality of cDNA of all the samples. The PCR conditions were a hot-start for 5 min at 94 °C; 35 cycles of 30 s at 94 °C, 30 s at 60 °C and 30 s at 72 °C; and 10 min at 72 °C.

qRT-PCR was performed using 100 ng cDNA, 12.5 μL of 2× UltraSYBR Mixture (CWBIO, Beijing, China) and 0.5 μM of each primer in a 25 μL volume. The specific primers used to amplify the GhLRP and tubulin transcripts were the same as those used for RT-PCR. Amplification was carried out in a LightCycler 480 II real-time PCR detection system (Roche, Penzberg, Germany). The PCR conditions were: 95 °C for 10 min, followed by 40 cycles of 95 °C for 15 s and 60 °C for 1 min. Independent biological triplicates and technical triplicates were used for each experiment. The 2^−ΔΔ^*^C^*^t^ method was used to calculate the expression levels of relevant genes.

### 4.6. Antiserum Production, Protein Extraction and Western Blot Analysis

The antibody was produced by Beijing Protein Innovation Company, Ltd. (Beijing, China; http://www.proteomics.org.cn/). A peptide corresponding to the amino acid sequence (EYKDKQDEDKEHKNEE) of *GhLRP* cDNA was synthesized, and 200 μg of the synthesized peptide was used to immunize rabbits subcutaneously. Booster shots were given at 12-day intervals with the same amount of antigen three times.

Soluble protein was extracted from 100 mg of mature kernel flour in 1 mL of extraction buffer (20 mM Tris-HCl, 5 mM ethylene diamine tetraacetic acid (EDTA), 20 mM NaCl, pH 8.0). The homogenate was centrifuged at 13,000× *g* for 15 min, and then, the suspension was used for western blot. About 20 μg of total soluble protein was separated by 12% SDS-PAGE and electroblotted onto polyvinylidene fluoride (PVDF) membranes (Millipore, Billerica, MA, USA) with an electrotransfer apparatus. Membranes were blocked in bovine serum albumin (BSA) blocking buffer (3% BSA in 1× Tris-buffered saline and Tween 20 (TBST), 50 mM Tris-HCl, 150 mM NaCl, 0.05% Tween 20, pH 7.5) at 4 °C overnight and then incubated with anti-GhLRP antibody (1:10,000) for 1 h. After washing the membranes three times for 15 min each with 1× TBST buffer, the goat anti-rabbit alkaline phosphate-conjugated IgG (Promega, Madison, WI, USA) as a secondary antibody (1:5000) was added into the 1× TBST buffer. Protein bands were visualized in alkaline phosphatase substrate buffer.

### 4.7. Lysine, Protein, Lipid and Starch Analyses

Twenty mature kernels (about 6 g) of transgenic and WT maize were ground to a powder for protein and lysine content analyses. Total nitrogen content was determined from about 0.1 g of maize flour using a classical Kjeldahl method. The conversion coefficient between nitrogen and protein content was 6.25. Technical and biological protein analyses were replicated three times.

Lysine content was quantified using the ninhydrin method. The ninhydrin reaction reagent was prepared by mixing A and B buffers. A buffer was prepared by dissolving 1 g ninhydrin in 25 mL of 95% alcohol solution, and the B buffer was prepared by dissolving 40 mg stannous chloride in 50 mL of 0.2 M sodium citrate buffer (pH 5.0). The reagent solution was stored away from light and prepared just before use. Lysine content analysis was performed according to the method of Beckwith [35], with some modifications. First, 1 mL ddH_2_O and 2 mL ninhydrin reaction reagent were added to a test tube containing 10 mg maize flour and mixed well. The test tube was then incubated in boiling water for 20 min. Next, 3 mL of 50% ethanol were added after the tube cooled to room temperature, and 2 mL of the mixture were removed to a 2 mL microfuge tube and centrifuged at 13,000× *g* for 10 min at room temperature. The absorbance of the supernatant at 570 nm was measured by spectrophotometer (Techcomp UV2300, Shanghai, China). The lysine concentration of each sample was determined by comparison to a standard curve established using 100 μg/mL leucine solution, and the lysine content was calculated using the following equation: Lys content (g/100 g dry weight) = (measured lysine content × hydrolysis volume)/sample weight. Independent biological triplicates and technical triplicates were used for each experiment. Lipid and starch contents were analyzed by near-infrared reflectance spectroscopy (NIRS) on a VECTOR22/N (Bruker, Karlsruhe, Germany) [36]. Independent biological triplicates and technical triplicates were used for each experiment.

### 4.8. Zein and Non-Zein Proteins Extraction and Quantification

The lipids in approximately 50 mg of maize flour were eliminated in 1 mL hexane for 1 h, with one repetition. Zein and non-zein proteins were extracted from the skimmed flour in 1 mL of extraction buffer containing 12.5 mM sodium borate (pH 10.0), 1% SDS and 2% β-mercaptoethanol, according to the method of Wallace and Larkins with some modifications [37]. After incubation with shaking at 37 °C up to overnight, the sample was centrifuged at room temperature for 15 min at 13,000× *g*, and the supernatant as a total protein extract was removed to a new 1.5 mL microfuge tube. To fractionate the zein proteins from other proteins, 700 μL of ethanol was added to the 300 μL of total protein extract, mixed well and incubated for 2 h. The sample was again centrifuged at room temperature for 15 min at 13,000× *g*; the supernatant contained the total zein fraction, while the pellet consisted of non-zein proteins. To check the zein proteins by SDS-PAGE, 5 μL samples were loaded to 15% polyacrylamide gel. For protein content analysis, the zein and non-zein proteins were dried in a SpeedVac (Christ, Osterode, Germany), and the pellets could be resuspended in 200 μL ddH_2_O. Protein concentration was estimated using a bicinchoninic acid (BCA) protein assay kit (Pierce, Rockford, IL, USA), according to the manufacturer’s instructions. First, 2.0 mL of working reagent were added to a test tube containing 100 μL of each BSA standard and sample and mixed well. Next, the test tube was incubated at 37 °C for 30 min. When all tubes were cooled to room temperature, the absorbance of all samples at 562 nm was measured by spectrophotometer (Techcomp UV2300, Shanghai, China). The protein concentration of each sample was determined by using the standard curve. For protein fraction analysis, each line was analyzed in triplicate.

### 4.9. Agronomic Quality Measurement

The impact of GhLRP protein accumulation on maize agronomic traits was estimated in each of the three transgenic lines. Each of the measured agronomic traits was replicated three times for the transgenic lines and wild-type (WT).

### 4.10. Statistical Analysis

Statistical analysis was performed using Microsoft Excel (Microsoft, Redmond, WA, USA). Results are presented as the mean ± standard deviation. For statistical analysis, a two-tailed unpaired *t*-test was performed to evaluate the difference in a given measured trait between the transgenic lines and WT (* *p* < 0.05; ** *p* < 0.01).

## 5. Conclusions

The lysine-rich protein gene, *GhLRP*, not only increased the lysine content of transgenic maize kernels, but also had no negative effect on plant quality or agronomic characters. This study demonstrated the feasibility of improving maize lysine content by introducing a lysine-rich protein gene and provided valuable material for Quality Protein Maize (QPM) breeding.

## Supplementary Information



## Figures and Tables

**Table 1.** Lysine and total protein contents of T_1_ kernels in 26 transgenic lines.

Line	Lysine content (g/100 g seed)	Protein content (g/100 g seed)
F12	0.37 ± 0.01*	12.36 ± 0.5*
F13	0.38 ± 0.01*	10.20 ± 0.3 ns
F17	0.35 ± 0.01*	9.93 ± 0.4 ns
F19	0.46 ± 0.02**	10.40 ± 0.3 ns
F28	0.39 ± 0.02*	10.38 ± 0.6 ns
F33	0.37 ± 0.03*	10.23 ± 0.2 ns
F34	0.35 ± 0.03*	9.54 ± 0.3 ns
F35	0.36 ± 0.01*	10.01 ± 0.2 ns
F38	0.40 ± 0.03*	9.89 ± 0.5 ns
F45	0.35 ± 0.03*	11.65 ± 0.7*
F47	0.37 ± 0.01*	11.79 ± 0.6*
F50	0.36 ± 0.02*	10.09 ± 0.2 ns
F52	0.35 ± 0.01*	9.79 ± 0.3 ns
F53	0.37 ± 0.02*	10.26 ± 0.4 ns
F57	0.36 ± 0.01*	10.47 ± 0.6 ns
F64	0.35 ± 0.01*	10.07 ± 0.2 ns
F72	0.37 ± 0.01*	10.18 ± 0.1 ns
F73	0.43 ± 0.02**	10.58 ± 0.6 ns
F77	0.38 ± 0.02*	10.32 ± 0.4 ns
F78	0.39 ± 0.01*	11.09 ± 0.6*
F83	0.37 ± 0.01*	10.50 ± 0.4 ns
F84	0.38 ± 0.02*	10.77 ± 0.6 ns
F87	0.36 ± 0.01*	11.47 ± 0.8*
F88	0.40 ± 0.01*	11.56 ± 0.7*
F89	0.37 ± 0.02*	9.75 ± 0.7 ns
F91	0.37 ± 0.01*	10.02 ± 0.4 ns
WT^a^	0.28 ± 0.01	10.19 ± 0.3

a The segregating populations of hybrid 08 × 178 in T_1_ were used as a control (WT);

* and ** indicate statistically significant differences between the transgenic lines and WT with *p* < 0.05 and *p* < 0.01 (Student’s *t-*test), respectively; ns = not significant; all the calculations were performed with technical triplicates and biological triplicates, and 20 mature kernels were used per assay.

**Table 2.** Lysine and protein contents of T_2_, T_3_ and T_4_ kernels in three transgenic lines.

Line	Lysine content (g/100 g seed)			Protein content (g/100 g seed)		
	
	T_2_	T_3_	T_4_	T_2_	T_3_	T_4_
F12	0.39 ± 0.02*	0.37 ± 0.01*	0.38 ± 0.01*	13.17 ± 0.5*	12.40 ± 0.1*	12.55 ± 0.2*
F78	0.38 ± 0.01*	0.39 ± 0.01*	0.40 ± 0.01*	12.31 ± 0.2*	12.29 ± 0.5*	13.15 ± 0.5**
F88	0.41 ± 0.01*	0.41 ± 0.02*	0.44 ± 0.02**	12.46 ± 0.4*	12.40 ± 0.2*	12.70 ± 0.1*
WT^a^	0.26 ± 0.02	0.30 ± 0.01	0.31 ± 0.01	9.93 ± 0.1	10.11 ± 0.2	10.20 ± 0.2

a The segregating populations of hybrid 08 × 178 in T_2_, T_3_ and T_4_ were used as a control (WT);

* and ** indicate statistically significant differences between the transgenic lines and WT with *p* < 0.05 and *p* < 0.01 (Student’s *t-*test), respectively; ns = not significant; all the calculations were performed with technical triplicates and biological triplicates, and 20 mature kernels were used per assay.

**Table 3.** Starch and lipid contents of T_2_ and T_3_ kernels in three transgenic lines.

Line	Lipid content (g/100 g seed)		Starch content (g/100 g seed)	
	
	T_2_	T_3_	T_2_	T_3_
F12	4.35 ± 0.31	4.04 ± 0.10	65.34 ± 0.95	69.61 ± 0.17
F78	4.68 ± 0.22	4.46 ± 0.25	65.82 ± 1.10	68.96 ± 1.69
F88	4.05 ± 0.14	4.32 ± 0.14	66.14 ± 0.54	71.69 ± 1.20
WT^a^	4.50 ± 0.50	4.03 ± 0.12	69.10 ± 0.32	72.25 ± 0.84

a The segregating populations of hybrid 08 × 178 in T_2_ and T_3_ were used as a control (WT); differences in agronomic traits between transgenic lines and WT are analyzed using a Student’s *t*-test; all the calculations were performed with technical triplicates and biological triplicates, and 20 mature kernels were used per assay.

**Table 4.** Agronomic traits of T_2_ kernels in three transgenic lines.

Line	PH (cm)	EH (cm)	EL (cm)	BTL (cm)	ED (cm)	ER	KW (g)
F12	213.6 ± 6.7	100.3 ± 4.5	9.5 ± 2.4	2.4 ± 1.4	40.6 ± 3.6	14	29.3 ± 6.1
F78	195.8 ± 5.5	98.2 ± 5.2	10.2 ± 2.3	0.5 ± 0.4	38.7 ± 3.9	16	26.8 ± 7.4
F88	200.5 ± 7.8	90.3 ± 3.2	7.9 ± 1.4	1.4 ± 1.2	37.2 ± 1.5	16	20.4 ± 0.3
WT^a^	203.5 ± 8.3	87.6 ± 5.4	11.3 ± 1.5	0.9 ± 0.6	42.3 ± 2.6	14	27.3 ± 2.0

a The segregating populations of hybrid 08 × 178 in T_2_ were used as a control (WT); PH, plant height; EH, ear height; EL, ear length; BTL, bald tip length; ED, ear diameter; ER, numbers of ear rows; KW, 100-kernel weight; values are the average measurements for each line in T_2_ progenies ± standard deviation; differences in agronomic traits between transgenic lines and WT were analyzed using a Student’s *t*-test.
