# Supplementary material for: Seed-Specific Expression of a Lysine-Rich Protein Gene, GhLRP, from Cotton Significantly Increases the Lysine Content in Maize Seeds
Source: Int J Mol Sci. 2014 Mar 27;15(4):5350–65. doi: 10.3390/ijms15045350 (PMC4013568; doi:10.3390/ijms15045350)
Supplement: Supplementary file 1 [file ijms-15-05350-s001.pdf]

Supplementary Information

Figure S1. Open reading frame and amino acid sequence of the *GhLRP* gene. (**A**) Open reading frame sequence of the *GhLRP* gene; (**B**) GhLRP protein sequence; (**C**) Amino acid composition of the GhLRP protein.

(A)

```
1     ATGATCGGTA GCCACACCGT CTCGTCAACG ACTCGACATT TATTCCAGAC ACAAACAACC
61    TCATCAGAGC TGCCACAATT GGCTTCAAAA TACAAAAAGC ACGAAGAGTC TGAATACAAA
121   CAGCCAAAAT ATCACGAAGA GCACCCAAAA CATGAGAAGC CTGAAATGCA CAAGGAGGAA
181   AGACAAAAAC CCTACAAACA ACATGAAGAG TACCACGAGT CACACGAATC GAAGAAGCAC
241   GAAGAGTACA ATAAAGACAA ACCCGATTTC CCCAAATGGG AAAAGCCTAA AGAGCACGAG
301   AAACACGAAG TCGAATATCC GAAAATACCC GAGTACAAGG ACAAACAAGA TGAGGATAAG
361   GAACATAAAA ATGAAGAGTT CCACGAATCA CGCGAATCAA AGGAGCACAA AGAGTATGAG
421   AAAGAAAAAC CCGAGTTCCC CAAACAGCAA AAGCCTAAAG AGCACGAGAA ACACCAAGTC
481   GAATATCCGG AAATAACCCA GTACAAGGAA GAGCAAGATA AGAGTAAGGA ACATAAAGAT
541   GAAGAGTGCC ACGAGTCACA CGAATCGAAA GAGCACGAAG AGTACTAG
```

(B)

```
1     MIGSHTVSST TRHLFQTQTT
21    SSELPQLASK YKKHEESEYK
41    QPKYHEEHPK HEKPEMHKEE
61    RQKPYKQHEE YHESHESKKH
81    EEYNKDKPDF PKWEKPKEHE
101   KHEVEYPKIP EYKDKQDEDK
121   EHKNEEFHES RESKEHKEYE
141   KEKPEFPKQQ KPKEHEKHQV
161   EYPEITQYKE EQDKSKEHKD
181   EECHESHESK EHEEY*
```

(C)

Amino acid		Num	%w/w
A	Ala	1	0.32
C	Cys	1	0.44
D	Asp	7	3.36
E	Glu	47	24.93
F	Phe	4	2.38
G	Gly	1	0.27
H	His	22	12.30
I	Ile	3	1.42
K	Lys	36	18.97
L	Leu	3	1.42
M	MET	2	1.08
N	Asn	2	0.95
P	Pro	14	5.81
Q	Gln	12	6.32
R	Arg	3	1.88
S	Ser	14	5.30
T	Thr	7	3.01
V	Val	3	1.27
W	Trp	1	0.74
Y	Tyr	12	7.84

Figure S2. Selection of maize resistant callus and plant regeneration. (**A**) Maize callus on selection medium containing 15 mg/L hygromycin; (**B**) Differentiation of resistant callus; (**C**) Regenerated plants in the rooting medium; (**D**) Regenerated plants in a flowerpot; (**E**) Regenerated plants in the field; (**F**) T_0 transgenic maize seeds.

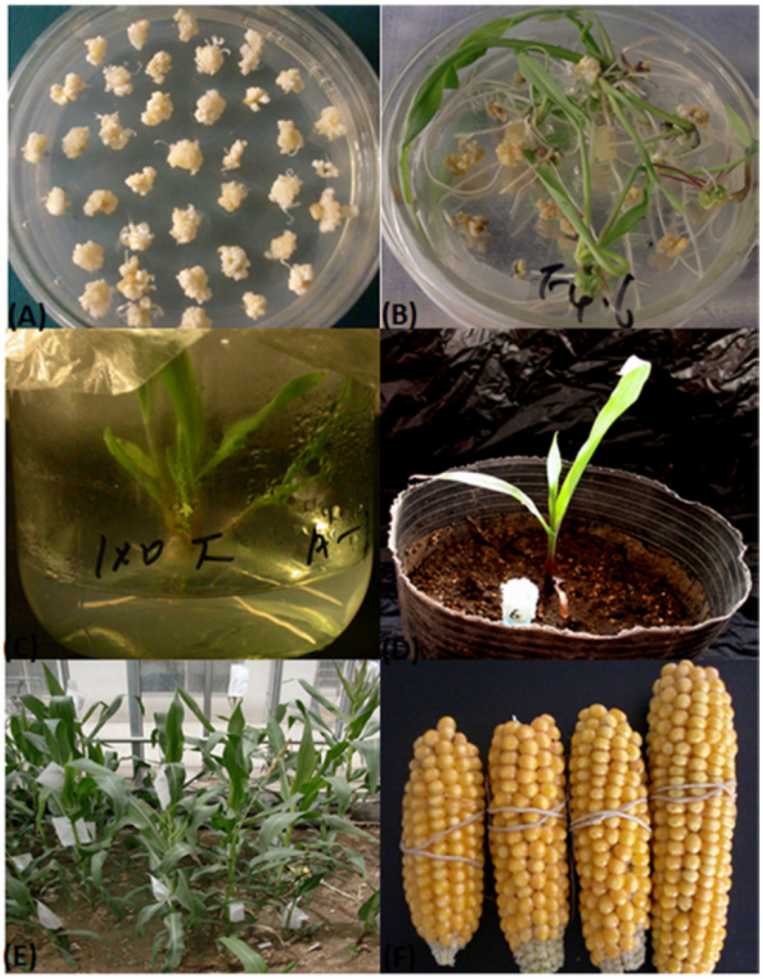

Figure S3. Zein and non-zein accumulation pattern in T_4 kernels of three transgenic lines. (**A**) Zein content; (**B**) Non-zein content. All the calculations were performed with technical triplicates and biological triplicates, and 20 mature kernels were used per assay. Student's *t*-test is used to evaluate the difference between the transgenic maize and WT (* $p < 0.05$; ** $p < 0.01$); (**C**) Zein proteins of WT, F12, F78, and F88 lines were analyzed by SDS–PAGE. For SDS–PAGE, 5 μL samples were loaded to 15% polyacrylamide gel. The size for each protein marker and sample band was indicated by the numbers in the "kDa" columns.

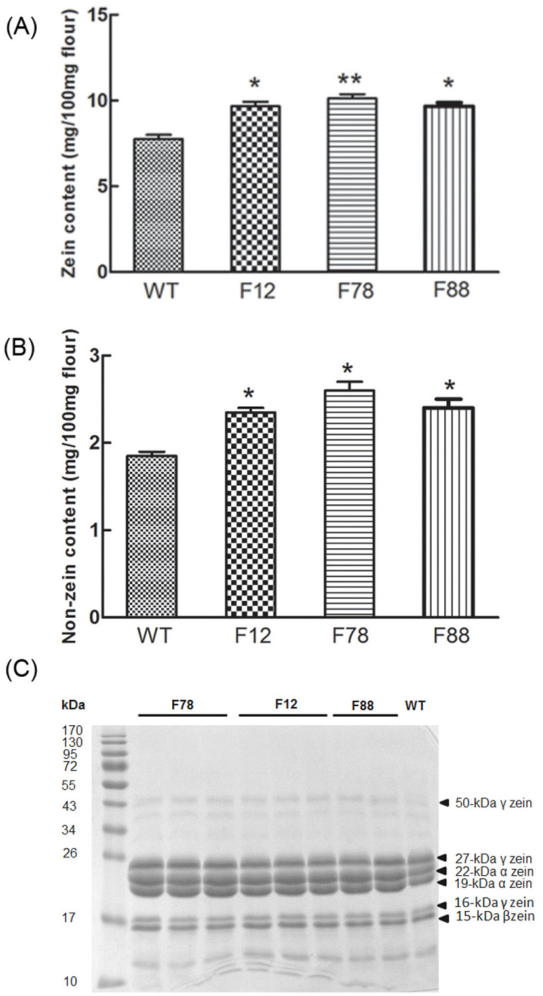

Table S1. Agronomic traits of T_3 kernels in three transgenic lines.

Line	PH (cm)	EH (cm)	EL (cm)	BTL (cm)	ED (cm)	ER	KW (g)
F12	200.7 ± 3.2	98.7 ± 5.4	11.8 ± 1.8	1.0 ± 0.7	37.7 ± 0.8	14	24.4 ± 0.9
F78	198.5 ± 7.5	89.3 ± 5.8	13.2 ± 1.6	0.4 ± 0.3	42.7 ± 1.2	16	25.2 ± 1.2
F88	210.3 ± 4.2	92.3 ± 4.2	12.0 ± 1.3	1.9 ± 1.4	39.2 ± 2.8	14	27.3 ± 3.4
WT [a]	202.5 ± 5.8	89.3 ± 3.8	11.3 ± 2.3	0.7 ± 0.6	39.8 ± 0.3	16	26.8 ± 4.0

[a] The segregating populations of hybrid 08 × 178 in T_3 were used as a control (WT). PH, plant height; EH, ear height; EL, ear length; BTL, bald tip length; ED, ear diameter; ER, number of ear rows; KW, 100-kernel weight. Values are average measurements for each line in T_2 progenies ± standard deviation. Differences in agronomic traits between transgenic lines and WT were analyzed using a Student's *t*-test.
